# Tonsillitis

**Published:** 1884-05

**Authors:** T. A. De Blois

**Affiliations:** Laryngologist to the Boston Dispensary; Assistant Physician to the Department for Diseases of the Throat and Nose, Boston City Hospital


### TONSILLITIS.*

BY T. A. DE BLOIS, M.D.,

*Laryngologist to the Boston Dispensary; Assistant Physician to the Department for Diseases of the Throat and Nose, Boston City Hospital.*

[New York Medical Journal.]

In bringing up again this, to say the least, "considerably discussed" subject, it is not with the belief that I have anything original to offer in either pathology, diagnosis, or treatment, but, as I have had the fortune, good or otherwise, to have been thrown almost daily with these cases, it appears to me that, by classing the results of a few years' treatment, an idea of its advisability might be gained.

I might, in order to illustrate the frequency of tonsillitis, in comparison with other affections coming to a special dispensary clinic, state that from the 1st of June, 1880, until the 1st of April, 1883—a space of nearly or during three years of almost daily treatment of diseases of the throat and nose at the Boston Dispensary—I saw 222 cases of tonsillitis in comparison with 2,008 cases, total of all kinds; in other words, eleven per cent. of the whole number of cases. As regards the distribution of these cases throughout the year, taking the year commencing April 1, 1882, to March 31, 1883, as a criterion, there were 819 cases, 110 being of tonsillitis, or 12½ per cent. In April, 77 total, 8 per cent. cases of tonsillitis. May, 63 cases, of which 8 per cent. were tonsillitis. June, 49 total cases, with 2 per cent. of tonsillitis. July, 60, with 20 per cent. of tonsillitis. August, 64, with 12 per cent of tonsillitis. September, 54, with 9 per cent of tonsillitis. October, 45, with 9 per cent. of tonsillitis. November, 44, with 20 per cent. December, 24 per cent. January, 26 per cent. February, 18 per cent. March, 7 per cent.

I regret that there has been no means of considering the different states of the weather as being a factor in the causation of tonsillitis; but, from careful observation, it appears to me that a season of damp weather, although warm, is more apt to be the time when we see more of tonsillitis than a season of dryer and colder weather. As regards the causation of this affection, it is extremely difficult, in so many cases that we see once and often not afterward, to say what has led to it.

*Read at the Section of Clinical Medicine of the Suffolk District Mass., Medical Society, March 12th, 1884.

The theory of a life in a vitiated and effete atmosphere, the presence of decaying animal substances in dissecting-rooms, etc., has been sufficiently written about to be familiar to every one; they may or may not be factors in its production, but certainly the larger part of the tonsillitis patients that we see are not exposed to these conditions, and are so much of their time exposed to the severities of a changeable climate that it would seem as if we ought to look more at the out-door than the in-door surroundings for at least the exciting causes. A low condition of the system, I think, without doubt, shows itself as predisposing the patient to this local glandular affection, just as it might to a series of furuncles, an abscess, or local lesion of the kind.

We come next to the point of to what class of diseases tonsillitis belongs I have always held the opinion, although at variance with that of the majority of authorities, that tonsillitis was not a constitutional disease. I have never seen anything to cause me to change my opinion. I believe the lesion to be entirely a local one, and I think the febrile and other symptoms are as easily accounted for on that ground as those occurring in acute coryza, burns, and other undoubtedly local affections. As to the course of the disease, I can speak with much less self-assertion. I am perfectly honest in my statement that the more I see of it the less I think I know about it. Certainly I never should think of dividing it into classes —as, for instance, a class that "walks" and a class that "stays at home." You may see a patient one day able to walk, and the next day, if you let him alone, he will not be able to walk; and others, if you let them alone, will be better than the day before. So also, as regards the abscess of the tonsil, or around the tonsil, some cases of tonsillitis go on to suppuration in spite of whatever you may do to the contrary; and others, that appear as if they were about to form an abscess, and which will last with great swelling and pain for two weeks and over, will then subside without your being able to find a drop of pus. So, would it not be wiser, or at least more honest, to say that local inflammations of the tonsils differ from each other in degree rather than in kind, and that, when a gland has suppurated, *then* we know it, and we did not know it before?

Now, I have said that tonsillitis in different cases differs in degree and also in appearances—probably caused by the original size and shape of the tonsil. There are some that appear to be entirely covered in by the reflection of the anterior pillar of the fauces, and then the swelling seems to be rather above and back of them; then, although the pain may be as great, breathing and deglutition will not be so much interfered with. Then there are other cases starting in a tonsil originally large, where the inflammation does not seem to greatly involve the soft palate, but the tumor will be lower; it will be more evident that it is in the gland itself, and there will be more follicular secretion on its surface. But it is the same tonsillitis.

As to diagnosis, it would be better to say differentiation, for, if you can exclude the exanthemata, some of the manifestations of syphilis, and, above all, the local symptoms of diphtheria, you would probably have tonsilltis left. And then there comes the point as to whether tonsillitis ever turns to diphtheria, and the converse. I believe tonsillitis and the local manifestations of diphtheria to be as distinct as I believe the local venereal sore and the primary lesion of syphilis to be, although there are many to whom this simile has no significance, but it is with great difficulty that they are invariably recognized; but when we think we have a case of tonsillitis, and suddenly find a bit of membrane in the larynx or posterior nares, it is much pleasanter to think that one disease runs into the other than to allow that there has been an unfortunate error in diagnosis.

Now, there is, besides these, a pharyngitis or tonsillitis without exudation, only erythema, which we sometimes find in the same house and generally in the same family, and sometimes synchronous with a case of diphtheria. What is this? Is it a lesser form of the same constitutional poisoning, or is it to be classed among the cases of idiopathic tonsillitis? Dr. McKenzie, from his vast experience, speaks of a form of diphtheria without exudation, and, as the membrane is only a local symptom of diphtheria, why may not some of the symptoms be found wanting in that as in other diseases?

About a year ago I was called to see a woman with throat symptoms. Upon examination, both tonsils and the velum were greatly swollen and of a dark-red color; both tonsils pretty well covered with a white exudation, which I was able to wipe off, leaving white points, through the middle of which the probe passed into the dilated mouths of the follicles; found nothing white in either posterior nares or larynx; the glands of the neck were greatly swollen; temperature 103°. No other symptoms. I believed I had a case of double tonsillitis, and told the friends so; treated with Dover's powder, etc. The next day the tonsils were about the same, but the nostrils contained a whitish matter, watery mucus issuing from them; felt pretty sure then I had a case of diphtheria; had to hedge a good deal with the inquiring friends. The third day the tonsils were not so bad; the nose was about the same, but externally there was a broad red flush over the bridge of the nose; there was no mistaking it. I couldn't hedge any more; it was one of those rare and severe cases of erysipelas commencing first in the fauces. So I told the family how much I had been mistaken, and the next day another physician was called. I could only console myself by thinking that the treatment had been all right, no matter how it turned out.

Fortunately, there are not many such puzzling cases, and the little point given by Dr. Jacobi—that the small white spots in tonsillitis always cover the orifice of a follicle, whereas in diphtheria they do not necessarily do so—shows us very well in most doubtful cases. The temperature, and the swelling or other condition of the glands of the neck, are of no use whatever; you may or you may not find them in either diphtheria or tonsillitis, and so it is in most of the other points of difference; they answer well enough for one, and they hold good for the other also.

The prognosis I suppose, is always good, although the duration of the disease is so very uncertain; they certainly almost always get well. The cases where an abscess has formed and bursts during sleep, strangling the patient, are, of course, well known to every one. There are cases where attacks of tonsillitis rapidly succeed each other, and where the tonsils do not go back to their normal state, gradually becoming hypertrophied together with a much congested condition. This state ought to be taken into consideration when speaking of the invariably favorable prognosis in tonsillitis.

The treatment of tonsillitis is perhaps, the hardest part of the subject to touch upon. Everybody who gets a case of tonsillitis treats it, and there are, generally speaking, as many modes of treatment as there are physicians who treat it. Like oysters, the tonsils are boiled steamed, or frozen, according as the medical attendant may fancy the hot gargle, hot spray, or ice treatment. Nothing can be said against these; it is all done with the one object in view of relieving the symptoms, and, of necessity, the treatment employed exemplifies the past experience and best judgment of the practitioner; what right has one to criticise the judgment any more than the taste of another?

The treatment which I have employed from time to time during the past few years in tonsillitis has most certainly been modified by my daily experience, but, although it has not always been what would please the most fastidious taste, still I have never hesitated to employ it in my own case when I thought I required it, and I can not say that I think I suffered greatly from the treatment. My first efforts were with astringents and escharotics. I used a good deal of iodine and nitrate of silver, and I do not believe that I got brilliant results; it would be absurd to say that I should have done better had I employed some other line of treatment, for that would be only conjecture. I combined this with the constitutional exhibition of iron and quinine, which seemed to have some good effect on the general malaise, not to speak of the slight local astringent effect. I never knew of any very protracted cases; I do not believe I shortened the course much—do not think I lengthened it.

Observing the tension of the parts during the first few days of some cases of tonsillitis, and believing, in many of these, the almost tetanic closure of the jaws was due to this tension, above all, remembering the relief afforded by the early incision of a furuncle, I adopted the plan of scarifying the tonsils with many shallow punctures; by this some little local bleeding was produced, and in almost all cases alleviation of pain; the jaws in some cases could be separated half an inch to an inch farther immediately after the operation. As far as I could judge, this treatment, combined with the use of tonics, has shortened the course of the affection a day or so. It seemed satisfactory, and in some cases I have continued the same treatment ever since. Guaiacum, combined with chlorate of potash and currant paste, I have found invaluable in the form of troches, as they do not taste very badly. Within the past year I have adopted the use of the Dover's powder—ten grains the first night, five the second, and five the third night, to be in each case followed by a saline cathartic in the morning; it does not always produce perceptible diaphoresis, it seems to relieve as much when it does not as when it does.

This treatment, followed by tonics I have found very good; it relieved some of the symptoms; it may have shortened the disease. I used this in ten out of eighty cases. On the whole, I believe I have had better results than from scarification, and, of course, it is better where the tension is not very great. Some years ago, having tonsillitis myself, I wanted to try the aconite treatment. I had no aconite at hand, but had some 1-50 gr. aconitine pills, which it seemed to me ought to do about as well. I used them often enough to produce severe toxic symptoms, but did not get the least relief to the tonsillitis. I have lately seen the aconite treatment in two cases; one was relieved and the other was not.

My principal objection to the use of aconite in dispensary practice is the great stupidity of the patients. It is a frequent occurrence, when prescribing for pharyngitis a gargle and a tonic, to have the patient return and acknowledge that he has swallowed the gargle and gargled with the tonic; now, under such conditions should we be justified in trusting such people with the use of so dangerous a remedy as aconite, which is generally prescribed by the drop? Hot water I have never used, except in catarrh and corysa, as an injection into the nose. I did not find it of much effect in these instances.

In conclusion, I can make these few deductions from my experience alone, and I only refer to my own experience. For a self-limited disease it is somewhat uncertain as to this limit; its diagnosis is generally very easy, but there are good opportunities for mistakes in some cases.

Different forms of treatment have had such different results in physicians' hands that it is wiser to make no suggestions, but leave each one to follow out his idea, or the result of his own experience.

